# Negative Self-Perception and Self-Attitude of Sexuality Is a Risk Factor for Patient Dissatisfaction Following Penile Surgery with Small Intestinal Submucosa Grafting for the Treatment of Severe Peyronie’s Disease

**DOI:** 10.3390/jcm8081121

**Published:** 2019-07-28

**Authors:** Armin Soave, Sebastian Laurich, Roland Dahlem, Malte W. Vetterlein, Oliver Engel, Timo O. Nieder, Peer Briken, Michael Rink, Margit Fisch, Philip Reiss

**Affiliations:** 1 Department of Urology, University Medical Center Hamburg-Eppendorf, 20246 Hamburg, Germany; 2 Institute for Sex Research, Sexual Medicine and Forensic Psychiatry, University Medical Center Hamburg-Eppendorf, 20246 Hamburg, Germany

**Keywords:** peyronie’s disease, penile induration, sexuality, patient satisfaction

## Abstract

Objective: To assess patient satisfaction with surgical outcome, body related self-perceptions, self-attitudes of sexuality, and health related quality of life after penile surgery with small intestinal submucosa (SIS) grafting for the treatment of severe Peyronie’s disease (PD). Material and methods: This retrospective study included 82 patients, who were treated with SIS grafting for severe PD between 2009 and 2013 at the University Medical Center Hamburg-Eppendorf. Patients were asked to complete standardized questionnaires including the International Index of Erectile Function Erectile Function domain (IIEF-EF), Short-Form (SF)-8 Health Survey, and Frankfurt Body Concept Scale-Sexuality (FKKS-SEX). Results: Follow-up was available in 58 (69.9%) patients. SIS grafting resulted in subjective straightening of the penis in 53 (91.3%) patients. After a mean follow-up of 28.9 ± 16.5 months, 24 (41.4%) patients were satisfied or very satisfied with surgical outcome. Postoperatively, the mean FKKS-SEX was 23.5 ± 5.9. In total, 36 (62.1%), 18 (31%), and four (6.9%) patients had FKKS-SEX scores corresponding to positive, neutral, and negative self-perception and self-attitude of sexuality, respectively. The mean postoperative SF-8 was 15.2 ± 6.4. Compared to the mean for German controls, patients achieved lower mean scores in the domains social functioning (50.4 ± 7.1), mental health (49.5 ± 9.2), and emotional roles (48.5 ± 6.8). Subjective shortening of the penis (Odds ratio (OR): 2.0), negative body related self-perceptions, and self-attitudes of sexuality (OR: 3.6) as well as IIEF-EF score (OR: 0.9) were risk factors for patient dissatisfaction (*p*-values ≤ 0.02). Conclusion: A relevant number of patients is not satisfied with surgical outcome after SIS grafting for the treatment of severe PD. Subjective shortening of the penis, negative body related self-perceptions, and self-attitudes of sexuality as well as IIEF-EF score were risk factors for patient dissatisfaction.

## 1. Introduction

Peyronie’s disease (PD) is a chronic connective tissue disorder of the tunica albuginea of the corpora cavernosa of the penis, and may cause relevant penile pain, penile plaque formation, and loss of penile length, as well as deformity and curvature of the penis [1]. As a consequence, PD may severely impair sexual activity and emotional wellbeing [2]. Up to 54% and 48% of PD patients present with erectile dysfunction (ED) [3] and depression [4], respectively, emphasizing the complexity of symptoms which patients may be affected by.

Surgical treatment is performed to correct penile deformity and penile curvature, with the aim to enable patients to have sexual intercourse. This represents the standard therapy in the stable phase of severe PD [5]. Various surgical procedures have been described, including tunical plication and grafting techniques [6,7]. Currently, xenogenic small intestinal submucosa (SIS) represents one of the most commonly used and widely established grafts for penile surgery in PD patients [5,7]. To date, there have been few published studies on SIS grafting for PD, and these studies have primarily focused on surgical outcome and complications [8,9,10,11], whereas patient reported treatment satisfaction has been considered in the minority of studies [12,13,14,15,16]. In addition, patient self-perceptions and attitudes of sexuality following SIS grafting have not been investigated so far.

Thus, the aim of the present study was to analyze body related self-perceptions and self-attitudes of sexuality as well as health-related quality of life following penile surgery with SIS grafting for severe PD, and to identify risk factors for patient dissatisfaction.

## 2. Patients and Methods

### 2.1. Patients

We retrospectively collected data of 82 patients with severe PD, who were treated with SIS grafting between 2009 and 2013 at the University Medical Center Hamburg-Eppendorf. Preoperative evaluation included an in-depth history of onset and duration of PD-specific symptoms, prior PD-specific treatment and general medical history. Auto-photographic documentation determined degree, direction, shape, and severity of penile curvature, as described in detail previously [17]. Physical examination and penile ultra-sonography determined location, number, and size of plaques of the tunica albuginea of the penis. Penile length was measured from suprapubic skin to distal glans in the stretched flaccid penis, as described previously [18]. Color duplex Doppler ultra-sonography (CDDU) of the penis combined with intracavernous injection of 20 µg Prostavasin was performed preoperatively according to established standard operating procedures [19].

### 2.2. Patient Reported Outcome Measure

Patient reported outcomes were assessed using a standardized questionnaire. Firstly, we employed several validated patient reported outcome measures. The validated International Index of Erectile Function Erectile Function domain (IIEF-EF) evaluated erectile function. No ED corresponded to an IIEF-EF score of 26–30, mild ED to a score of 22–25, mild to moderate ED to a score of 17–21, moderate ED to a score of 11–16, and severe ED to a score of 6–10. Health related quality of life was assessed with the validated Short-Form (SF)-8 Health Survey, consisting of eight dimensions including social functioning, mental health, emotional roles, role physical, bodily pain, general health, vitality, and physical functioning [20]. SF-8 physical component score and SF-8 mental component score were calculated weighting each SF-8 item using the norm-based scoring method as described previously [21]. The mean scores in the 8 dimensions were compared to the mean for German controls [22]. A score of 50 is the mean for the German general population, a higher score indicates increased quality of life. Body related self-perceptions and self-attitudes of sexuality were assessed with the Frankfurt Body Concept Scale-Subscale Sexuality (FKKS-SEX), consisting of eight items with six answering options, respectively. The subscale is intended to measure how satisfied patients are with their sexuality, how attractive they consider themselves to potential sexual partners, and how they deal with sexual intimacy. The maximum FKKS-SEX score is 36, the minimum six; a score of 6–18, 19–23 and 24–36 is indicating a negative, neutral, and positive self-perception and self-attitude of sexuality, respectively [23]. Secondly, we included non-validated questions on patient satisfaction, penile paresthesia, and sexual activity. Non-validated questions were assessed using a five-point Likert-scale.

### 2.3. Follow-Up

Patients were seen at various time points after surgery at the outpatient clinic of our institution and received the standardized questionnaire. In addition, physical examination, penile ultra-sonography, penile length measurement, and CDDU of the penis were performed. There was no preoperative evaluation of health-related quality of life and no preoperative evaluation of self-perception and self-attitude of sexuality. Missing baseline data is denoted in the respective tables of the results section of the manuscript.

### 2.4. Surgical Procedure and Postoperative Management

Generally, SIS grafting was performed under general anesthesia as described in detail previously [5]. In brief, a circumcision was performed, followed by complete de-gloving of the penis and careful exposure of the dorsal neurovascular bundle. Then, an artificial erection was achieved with intracavernous injection of sodium chloride to identify the maximum convexity of the penile curvature. A transverse incision of the tunica and/or the plaque was performed at the maximum convexity, and the lateral margins of the incision were extended in a Y-formed shape. The length of the Y-shape was chosen depending on the degree of the lateral penile curvature. The size of the SIS graft was chosen depending on the size of the resulting defect of the tunica albuginea of the corpora cavernosa. The Biodesign^®^ four-layer SIS (Cook Medical LLC, Bloomington, IN, USA) was transplanted to the defect and fixed to the tunica albuginea with 3-0 monofil continuous sutures. Then, an artificial erection was provoked again to control for complete straightening of the penis. In cases of remaining slight curvature, a tunical plication was performed according to Yachia’s technique [24] at the discretion of the surgeon. After closure of Buck’s fascia and skin, a suprapubic catheter was placed, and a compression bandage was put on the penis. Roland Dahlem, Margit Fisch, and Oliver Engel performed all surgical procedures.

Generally, the compression bandage and suprapubic catheter were removed on postoperative day five, and patients were discharged. Patients were advised to perform penile rehabilitation with daily stretching of the penis using a vacuum device plus daily intake of phosphodiesterase-5 inhibitors. Patients were not allowed to have sexual intercourse for six weeks postoperatively.

### 2.5. Statistical Analysis

All analyses were performed with SPSS 20 (SPSS Inc., IBM Corp., Armonk, NY, USA). All tests were two-sided and a *p* < 0.05 was set to be statistically significant. Differences between continuous variables in one group were assessed using the T-test. Differences between categorical variables were assessed with the Chi square test. Uni-variable binary logistic regression analysis was employed to identify risk factors for patient dissatisfaction. For uni-variable binary logistic regression analysis, patients were grouped as “satisfied” (patients, who responded that they were “satisfied” or “very satisfied” with surgical outcome) and “dissatisfied” (patients, who responded “undecided”, “dissatisfied”, or “very dissatisfied” with surgical outcome).

## 3. Results

### 3.1. Patient Characteristics

Table 1 presents clinical features of the patients. Hypertension, Morbus Dupuytren, and prostatic hyperplasia were the most common comorbidities in 25 (30.5%), 15 (18.3%), and nine (11%) patients, respectively. In total, 11 (13.4%) patients reported previous penile trauma. The mean degree of penile curvature was 65°, and the majority of patients reported dorsal and lateral-left curvature in 51 (62.2%) and 11 (13.4%) patients, respectively.

### 3.2. Patient Reported Outcomes

Follow-up was available in 58 (69.9%) patients. After a mean follow-up of 28.9 ± 16.5 months, complete straightening of the penis was achieved in 53 (91.3%) patients, while five (8.7%) patients reported insufficient straightening. Overall, 41 (70.7%) patients reported paresthesia of the penis, corresponding to hypoesthesia and hyperesthesia in 34 (82.9%) and seven (17.1%) patients, respectively. Penile paresthesia was not bothering in 23 (56.1%) patients. In total, 56 (96.6%) patients reported subjective shortening of the penis. There was no significant difference in measured penile length preoperatively and postoperatively (*p* = 0.9). Postoperatively, 36 (62.1%) patients reported subjective deterioration of erectile function. According to the IIEF-EF score, postoperatively, 22 (37.9%) and 13 (22.4%) patients had severe and moderate ED, respectively, compared to 11 (19.0%) and seven (12.1%) patients with severe and moderate ED preoperatively (*p* = 0.041; Table 2).

The mean postoperative FKKS-SEX was 23.5 ± 5.9, which corresponds to neutral to positive self-perception and self-attitude of sexuality. In total, 36 (62.1%), 18 (31%), and four (6.9%) patients had FKKS-SEX scores corresponding to positive, neutral, and negative self-perception and self-attitude of sexuality, respectively. The mean postoperative SF-8 was 15.2 ± 6.4. Compared to the mean for German controls, patients achieved lower mean scores in the domains social functioning (50.4 ± 7.1), mental health (49.5 ± 9.2), and emotional roles (48.5 ± 6.8); higher mean scores in the domains role physical (50.0 ± 6.8), bodily pain (54.5 ± 9.1), general health (51.5 ± 7.2), and vitality (51.5 ± 8.1); and equivalent mean scores in the domain physical functioning (49.0 ± 8.2).

### 3.3. Patient Satisfaction

In total, 24 (41.4%) patients were satisfied or very satisfied with surgical outcome, while 26 (44.8%) patients were dissatisfied or very dissatisfied. Altogether, eight (13.8%) patients were undecided regarding satisfaction with surgical outcome.

In total, 26 (44.8%) of 58 patients with postoperative subjective shortening of the penis were dissatisfied or very dissatisfied with surgical outcome. Overall, 17 (77.3%) patients of 22 patients with severe postoperative ED were dissatisfied or very dissatisfied with surgical outcome. In total, four (100%) patients with low body related self-perceptions and self-attitudes of sexuality were dissatisfied or very dissatisfied with surgical outcome. Of the 26 patients, who were dissatisfied or very dissatisfied with surgical outcome, 19 (73.1%), 19 (73.1%), and four (15.4%) patients had subjective shortening of the penis, subjective deterioration of erectile function, and low body related self-perceptions and self-attitudes of sexuality, respectively.

In uni-variable logistic regression analysis, subjective shortening of the penis, negative body related self-perceptions and self-attitudes of sexuality as well as IIEF-EF score were risk factors for patient dissatisfaction (*p*-values ≤ 0.02; Table 3).

## 4. Discussion

We found that 41% of patients were satisfied, whereas almost 45% of patients were not satisfied with outcome following surgery, although SIS grafting resulted in complete straightening of the penis in more than 90% of patients. Previously, others have reported inconsistent findings on patient satisfaction with surgical outcome following SIS grafting. Some studies found high satisfaction rates of 82–89% [13,14,15,16], while Chung et al. reported that more than 65% of patients were not satisfied with surgical outcome [12]. Variable findings among studies regarding patient satisfaction with surgical outcome may be due to differences in patient characteristics, follow-up, study design, and methods. For example, the prospective study by Sayedahmed et al. included 43 patients, who were recruited from two centers over a time period of eight years. Kovac et al. and Chung et al. included 36 and 46 patients, respectively, who were recruited over a time period of six years and also received dermal and synthetic grafts [12] or dermal and cadaveric pericardial grafts [13], which may render comparison of results difficult. Morgado et al. focused on patient satisfaction with sex life after surgery [16]. Other studies reported less subjective penile shortening in 5–71% of patients [12,13,14,15,16], compared to almost 97% of patients in the present study. Importantly, we could demonstrate that subjective shortening of the penis was a risk factor for patient dissatisfaction, which therefore could be a reason for the observed differences in patient satisfaction. In addition, the present study included a higher proportion of patients with moderate and severe preoperative ED, compared to 7–10% preoperative ED in previous studies [14,15]. It is well established that ED may deteriorate after SIS grafting [5]. We could demonstrate that subjective worsening of erectile function was not a risk factor for patient dissatisfaction. However, higher IIEF-EF postoperative scores significantly reduced the risk of dissatisfaction. Moreover, other studies evaluated patient satisfaction with 5-point scales [12], 4-answering possibilities [15], 3-answering possibilities [13], a modified Erectile Dysfunction Inventory of Treatment Satisfaction [16], or did not report in detail on how patient satisfaction was measured [14], which may contribute to variable results. Finally, we found a higher proportion of patients with penile paresthesia following SIS grafting, compared to previous reports [12,13,14,15,16]. These differences may rely on variable evaluation of penile sensibility among different studies. In addition, circumcision was performed in all patients in the present study and might have contributed to penile paresthesia. Moreover, the type of graft may play a role, although Kovac et al. did not report relevant differences in penile hypoesthesia among patients, who received SIS grafts, dermal grafts or Tutoplast grafts [13]. Finally, the dissection of the dorsal pedicle may also influence sensory changes. The majority of patients in the present study reported that penile paresthesia was not bothering. However, we cannot exclude that this may have contributed to overall dissatisfaction. In the present study, circumcision was performed in all patients during penile surgery, although a case series questioned its need in patients undergoing penile surgery for PD [25]. Although circumcision is in general considered a safe procedure, it may have adverse effects, e.g., hypoesthesia of the glans, as well as negative psychological consequences [26]. Thus, we cannot exclude that circumcision may have added to patient dissatisfaction.

For the first time, the present study incorporated patients’ body related self-perceptions and self-attitudes of sexuality, which was assessed with validated FKKS-SEX. Thus far, FKKS-SEX was primarily used in patients with psychiatric disorders, e.g., depression and addiction [23]. We found that the majority of patients had a positive or neutral self-perception and self-attitude of sexuality. Importantly, negative self-perception and self-attitude of sexuality was a risk factor for patient dissatisfaction. Thus, FKKS-SEX may represent a promising tool to preoperatively identify patients, who are at risk for dissatisfaction. At best, it may be helpful in detecting patients with relevant underlying psychological conditions prior surgery. Then, these patients may benefit from rigorous sexological evaluation, support, or intervention in the preoperative and postoperative setting. We used the validated SF-8 to assess health related quality of life, and found that patients had lower social functioning, mental health, and emotional roles compared to the mean for German controls, although neither SF-8 physical component score nor SF-8 mental component score were risk factors for patient dissatisfaction with surgical outcome. Thus far, SF-8 has mainly been used to evaluate patients with other chronic illness, e.g., migraine, depression, and diabetes [20]. Our findings indicate that this questionnaire may be useful in PD patients treated with SIS grafting. However, further prospective studies are needed to confirm the potential of FKKS-SEX and SF-8 in outcome measurement in these patients; and to analyze the possibility of identifying patients with underlying psychological conditions, who may benefit from sexological support or intervention.

We found considerable discrepancy between patients’ complaints and objective outcome. First of all, almost 97% of patients reported loss of penile length, whereas measurement revealed no significant change between preoperative and postoperative penile length. This corresponds to findings of other authors, who found that 71% of patients reported subjective loss of penile length, whereas measurement revealed penile shortening in 14% of patients [15]. These findings highlight the importance of thorough preoperative counseling, with the aim to lower patients’ unrealistic expectations of SIS grafting, such as restoration of penile length as it was prior disease onset. Second, 62% of patients reported subjective worsening of erectile function. Indeed, postoperatively, more patients reported severe and moderate ED. This corresponds to findings of other authors, who found that 32% of patients reported decreased rigidity, whereas only 18% of patients had IIEF scores corresponding to moderate and severe ED [15]. Compared to the present study, others have previously reported better [13,14,15] or worse postoperative erectile function [12]. Inconsistent findings regarding postoperative erectile function may be due to differences in methods, follow-up and patient characteristics. For example, other studies used IIEF-5 questionnaire [12,13,14,15,16], had longer [12,14,16] or shorter [13,15] follow-up or did not report on relevant comorbidities [14], which may be associated with ED. Importantly, with longer time from penile surgery, other factors like age and comorbidities may occur and have additional negative impact on erectile function. For example, increasing age is a well-established risk factor for erectile dysfunction [27].

The present study has important limitations, which are first and foremost inherent to the retrospective study design. The number of patients is low, and follow-up was not available in 30% of patients. Therefore, selection bias could have influenced the results both at baseline and at follow-up. In addition, preoperative or postoperative data, e.g., penile length measurement, was not available in a relevant number of patients. Particularly, this may have introduced relevant reporting bias. In addition to validated instruments, the questionnaire included non-validated questions, which renders comparability with results of other studies difficult. Neither FKKS-SEX nor SF-8 have been validated for the use in patients with PD. Moreover, patients did receive the questionnaire at different time points following SIS grafting, which might cause relevant heterogeneity of results. The effect of SIS grafting on health-related quality of life as well as self-perception and self-attitude of sexuality remains uncertain, since preoperative data on SF-8 and FKKS-SEX was not available. The incision of the tunica albuginea of the corpora cavernosa may also have an impact on outcome, especially erectile function, but was not assessed in the present study. Data on treatment of ED after SIS grafting was missing. Multivariable analysis to identify independent predictors for patient dissatisfaction was not possible, since the number of events was too low. Nevertheless, our data suggest that patient related features as subjective shortening of the penis, negative body related self-perceptions and self-attitudes of sexuality as well as IIEF-EF score were risk factors for patient dissatisfaction. Thus, validation of our results in prospective studies with larger patient cohorts is warranted.

## 5. Conclusions

A relevant number of patients with severe PD are not satisfied with surgical outcome after SIS grafting. The majority of patients have positive and neutral self-perception and self-attitudes of sexuality. Following SIS grafting, patients have lower social functioning, mental, and emotional health compared to controls. Subjective shortening of the penis, negative body related self-perceptions, and self-attitudes of sexuality as well as IIEF-EF score are risk factors for patient dissatisfaction. Further prospective studies with larger patient cohorts are necessary to validate these findings.

## Figures and Tables

**Table 1:** Clinical characteristics of 82 patients treated with small intestinal submucosa grafting for Peyronie’s disease.

	All (*n* = 82)
**Age** (years; mean (95% CI))	56.9 (55.5–58.5)
**Body mass index** (mean (95% CI)) 6 (7.3%) patients missing	26.6 (25.7–27.4)
**Comorbidities** (*n*; %)	
Hypertension	25 (30.5)
Diabetes	7 (8.5)
Depression	5 (6.1)
Morbus Dupuytren	15 (18.3)
Morbus Ledderhose	1 (1.2)
Prostatic hyperplasia	9 (11.0)
Penile trauma	11 (13.4)
**Smoking status** (*n*; %)	
Active	45 (54.9)
No	17 (20.7)
Unknown	20 (24.4)
**Penile curvature** (degree; mean, (95% CI)) 18 (22%) patients missing	64.8 (59.1–70.5)
**Direction of penile curvature** (n; %)	
Ventral	3 (3.7)
Dorsal	51 (62.2)
Lateral left	11 (13.4)
Lateral right	6 (7.3)
**Plaque size** (cm; mean, (95% CI)) 44 (53.7%) patients missing	2.9 (1.7–4.2)
**Duration of PD-specific symptoms** (days; mean (95% CI)) 1 (1.2%) patient missing	343.8 (217.7–469.8)
**Previous PD-specific treatments** (*n*; %)	
Potaba	26 (41.3)
Vitamine E	13 (20.6)
Steroids	1 (1.6)
Interferon	1 (1.6)
Verapamil	1 (1.6)
ESWT	1 (1.6)
None	24 (38.1)
Unknown	19 (23.2)
**Preoperative Resistance index** as measured by CDDU (mean (95% CI)) 29 (35.4%) patients missing	0.87 (0.82–0.93)

Abbreviations: CDDU = Color duplex Doppler ultra-sonography; CI = Confidence interval; ESWT = extracorporal shock wave therapy; PD = Peyronie’s disease.

**Table 2:** Change in preoperative and postoperative erectile function in 58 patients treated with small intestinal submucosa grafting for Peyronie’s disease.

	Preoperative	Postoperative	Difference	*p*-Value
**IIEF-EF score** (mean (95% CI))	12.2 (8.4–15.9) 29 (50%) patients missing	14.5 (12.2–16.9)	2.0 (−4.5–8.5)	0.13 *
**Erectile dysfunction according to IIEF-EF** (*n*; %)				0.041 ^#^
Severe	11 (19.0)	22 (37.9)		
Moderate	7 (12.1)	13 (22.4)		
Mild to moderate	4 (6.9)	6 (10.3)		
Mild	3 (5.2)	7 (12.1)		
No	2 (3.4)	10 (17.2)		
Missing	29 (50.0)	0 (0)		

* Paired samples T-test. ^#^ Chi square test. Abbreviations: CI = Confidence interval; IIEF-EF = international index of erectile function erectile function domain.

**Table 3:** Uni-variable logistic regression of subjective penile shortening, subjective reduced erectile function, negative body related self-perceptions and self-attitudes of sexuality, International Index of Erectile Function Erectile Function domain (IIEF-EF) and Short-Form (SF)-8 score predicting patient dissatisfaction with surgical outcome in 58 patients treated with small intestinal submucosa grafting for Peyronie’s disease.

	Odds Ratio	95% CI	*p*-Value
Subjective loss of penile length	2.026	1.152–3.563	0.014
Subjective reduced erectile function	2.154	0.507–9.147	0.298
Negative body related self-perceptions and self-attitudes of sexuality	3.632	1.231–10.718	0.020
IIEF-EF score	0.870	0.805–0.940	<0.001
SF-8 physical component score	0.963	0.898–1.034	0.299
SF-8 mental component score	0.971	0.920–1.026	0.298

Abbreviations: CI = Confidence interval; IIEF-EF = International index of erectile function erectile function domain; SF-8 = Short-Form-8 Health Survey.
